# Taltirelin is a superagonist at the human thyrotropin-releasing hormone receptor

**DOI:** 10.3389/fendo.2012.00120

**Published:** 2012-10-09

**Authors:** Nanthakumar Thirunarayanan, Bruce M. Raaka, Marvin C. Gershengorn

**Affiliations:** Laboratory of Endocrinology and Receptor Biology, National Institute of Diabetes and Digestive and Kidney Diseases, National Institutes of HealthBethesda, MD, USA

**Keywords:** taltirelin, thyrotropin-releasing hormone, human TRH receptor

## Abstract

Taltirelin (TAL) is a thyrotropin-releasing hormone (TRH) analog that is approved for use in humans in Japan. In this study, we characterized TAL binding to and signaling by the human TRH receptor (TRH-R) in a model cell system. We found that TAL exhibited lower binding affinities than TRH and lower signaling potency via the inositol-1,4,5-trisphosphate/calcium pathway than TRH. However, TAL exhibited higher intrinsic efficacy than TRH in stimulating inositol-1,4,5-trisphosphate second messenger generation. This is the first study that elucidates the pharmacology of TAL at TRH-R and shows that TAL is a superagonist at TRH-R. We suggest the superagonism exhibited by TAL may in part explain its higher activity in mediating central nervous system effects in humans compared to TRH.

## INTRODUCTION

Based on evidence that thyrotropin-releasing hormone (TRH) modulates a number of central nervous system (CNS) activities including arousal, antidepressant activity, anxiolytic effects, increase in locomotor activity, antagonism of pentobarbital-induced sedation, thermoregulation, and cardiovascular and gastrointestinal autonomic functions (Horita et al., 1986; Horita, 1998; Khomane et al., 2011), many analogs of TRH were synthesized and studied. Taltirelin (TAL) hydrate [(1-methyl-(S)-4,5-dihydroorotyl)-histidyl-prolinamide, TA-0910] is an analog that showed improved CNS activity (Suzuki et al., 1990; Yamamura et al., 1991, 1997) and lower thyrotropin (TSH)-releasing activity (Yamamura et al., 1997) compared to TRH in rodents. Based on these characteristics, TAL was studied as a treatment for neurodegenerative disorders and is the only TRH analog that has been approved for use in humans; it is used in Japan to treat patients with adult spinal muscular atrophy (Ceredist^®^).

In some mammals, including rodents, there are two subtypes of G protein-coupled (or seven-transmembrane-spanning) receptors for TRH. These are TRH receptor 1 (TRH-R1), which is the primary (or only) receptor in TSH-secreting cells, and TRH-R2, which is expressed throughout the CNS along with TRH-R1 but typically in different areas of the brain (O’Dowd et al., 2000; Sun et al., 2003). In humans, by contrast, only a single type of receptor for TRH, TRH-R, is expressed that is more similar to TRH-R1 than TRH-R2 (Duthie et al., 1993; Matre et al., 1993). It has been reported that TAL binds with lower affinity than TRH to receptors in rat or mouse pituitary and in brain tissue preparations (Kinoshita et al., 1997; Asai et al., 1999, 2005). However, the pharmacology of TAL at TRH-R has not been characterized.

In this study, we characterized TAL binding and signaling by TRH-R in a model cell system and show that TAL is a superagonist at TRH-R.

## MATERIALS AND METHODS

### MATERIALS

Dulbecco’s modified Eagle’s medium (DMEM) and fetal bovine serum were purchased from Biosource (Rockville, MD, USA). TRH (pyroGlu-His-ProNH_2_) and MeTRH (pGlu-His(1(τ)-methyl)-ProNH_2_) were purchased from Sigma (St. Louis, MO, USA). [^3^H]MeTRH was purchased from PerkinElmer (Waltham, MA, USA). TAL (N-[[(4S)-Hexahydro-1-methyl-2,6-dioxo-4-pyrimidinyl]carbonyl]-L-histidyl-L-prolinamide) was obtained from Tocris (San Diego, CA, USA).

### CELL CULTURE AND GENERATION OF CELLS STABLY EXPRESSING TRH-R

HEK-EM 293 (human embryonic kidney) cells stably expressing TRH-R were generated as follows. The human TRH-R cDNA in pcDNA3.1(+) was obtained from the Missouri S&T cDNA Resource Center (Rolla, MO, USA) and was subcloned into the pcDNA3.1(+)/hygromycin vector. HEK-EM 293 cells were transfected with the cDNA of TRH-R using FuGENE 6 transfection reagent (Roche Diagnostics GmbH, Mannheim, Germany) and the cell clones stably expressing TRH-R were selected using hygromycin (250 μg/ml). HEK-EM 293 cells stably expressing TRH-R were grown in DMEM containing 10% fetal bovine serum, 100 U/ml penicillin, 10 μg/ml streptomycin, and 200 μg/ml hygromycin B (Invitrogen, Carlsbad, CA, USA) at 37°C in a humidified 5% CO_2_ incubator.

### COMPETITION BINDING

**FIGURE 1:**
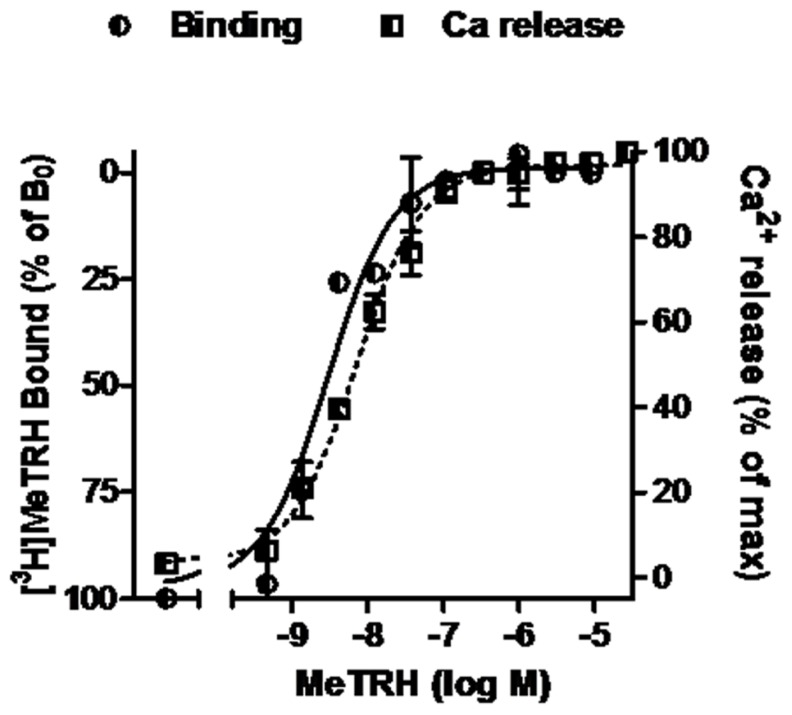
**MeTRH binding and signaling via Ca^2+^ in cells expressing TRH-Rs.** Competition binding with [^3^H]MeTRH and changes in cytosolic free Ca^2+^ concentration (Ca^2+^ release) were performed in intact HEK-EM 293 cells stably expressing TRH-Rs as described in Section “Materials and Methods.” The competition binding data are presented as the % of [^3^H]MeTRH bound to cells in the absence of unlabeled ligand (B_0_) and represent the results from three experiments conducted with duplicate measurements. Note the left axis is inverted. Intracellular Ca^2+^ release data are shown as a % of the maximal Ca^2+^ increase over basal. The curves represent the non-linear regression analyses of data obtained in one of three experiments with triplicate measurements using a sigmoidal dose–response method.

Competition binding assays were performed in monolayers of intact HEK cells expressing TRH-Rs. The cells (220,000 cells/well in 24-well plates) were preincubated with various concentrations of unlabeled TAL, TRH, or MeTRH for 15 min before addition of radioligand and then incubated at 37°C for 1 h with 4 nM [^3^H]MeTRH as described elsewhere (Engel et al., 2006). Non-specific binding was determined in incubations with excess non-radiolabeled MeTRH. IC_50_ is the concentration of unlabeled ligand that reduces specific binding of [^3^H]MeTRH by 50%. The receptor number per cell was calculated from competition binding curves of various doses of unlabeled MeTRH and 4 nM [^3^H]MeTRH (**Figure 1**) and found to be 16,000/cell.

### MEASUREMENT OF INTRACELLULAR CALCIUM MOBILIZATION

Cells stably expressing TRH-R were seeded in black-walled, clear-bottomed 96-well plates (Corning, NY, USA) at a density of 60,000 cells/well in DMEM with 10% fetal bovine serum and incubated for 24 h at 37°C in 5% CO_2_. The following day, the culture media was replaced with 100 μl of Hank’s balanced salt solution with 20 mM HEPES, pH 7.5 and the cells were loaded with 100 μl of calcium 4 fluorescent dye (Molecular Devices, Sunnyvale, CA, USA) for 1 h at room temperature before addition of compounds. Transient changes in intracellular [Ca^++^] induced by TAL, TRH, or MeTRH were measured using the FLIPR^TETRA^ system (Molecular Devices, Sunnyvale, CA, USA). Changes in fluorescence were detected at the emission wavelength ranges from 515 to 575 nm. The agonistic responses of ligands were assessed immediately upon their addition in a concentration range from 0.1 nM to 30 μM. Responses were measured as peak fluorescent intensity minus basal fluorescent intensity at each compound concentration and are presented as % of the maximum response.

### MEASUREMENT OF IP1 PRODUCTION

Cells were seeded at 220,000/well in white, solid bottom, tissue culture-treated 24-well plates and cultured at 37°C with 5% CO_2_ overnight. Serial dilutions of TAL, TRH, or MeTRH, in Hank’s balanced salt solution with 20 mM HEPES and 50 mM LiCl, pH 7.4, were added at 200 μl/well on the second day. After 60 min incubation at 37°C in 5% CO_2_, inositol monophosphate (IP1) content was measured using the IP-One ELISA kit (Cisbio International, France) according to the manufacturer’s protocol. The results were calculated as IP1 nanomoles/well and are presented as % of the maximum response.

### DATA ANALYSIS

The dose–response data were analyzed by non-linear regression of curve fit with one-site competition using GraphPad Prism software version 4 (GraphPad, Inc., San Diego, CA, USA) and the significance was determined by *t*-test or ANOVA.

## RESULTS

**FIGURE 2:**
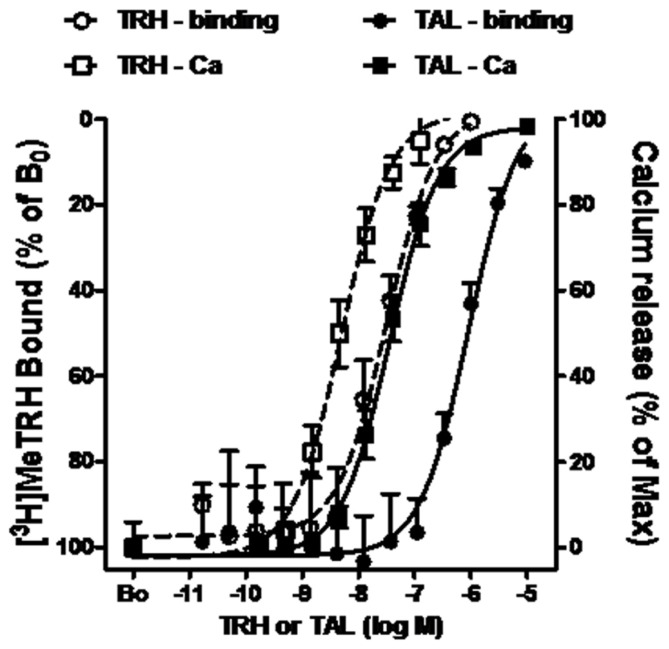
**TAL and TRH binding and signaling via Ca^2+^ in cells expressing TRH-Rs.** Competition binding with [^3^H]MeTRH and changes in cytosolic free Ca^2+^ concentration (Ca^2+^ release) were performed in intact HEK-EM 293 cells stably expressing TRH-Rs as described in the legend to **Figure 1**. The competition binding data are presented as the % of [^3^H]MeTRH bound to cells in the absence of unlabeled ligand (B_0_) and represent the results from three experiments conducted with duplicate measurements. Note the left axis is inverted. Intracellular Ca^2+^ release data are shown as a % of the maximal Ca^2+^ increase over basal. The curves represent the non-linear regression analysis of data obtained in three experiments with duplicate or triplicate measurements using a sigmoidal dose–response method.

The pharmacology of TAL binding and signaling was studied in HEK-EM 293 cells, which do not endogenously express TRH-Rs, engineered to express 16,000 TRH-Rs/cell (**Table 1**). In competition binding assays using 4 nM [^3^H]MeTRH and various doses of unlabeled MeTRH, the concentration of MeTRH that half-maximally inhibited [^3^H]MeTRH binding (IC_50_) was 3.0 nM (**Figure 1**). The half-maximally effective concentration (EC_50_) of MeTRH for stimulating an increase in cytosolic Ca^2+^ concentration (Ca^2+^ release) was 7.2 nM, which is not different from the IC_50_ (*p* > 0.1). Thus, MeTRH is an agonist at TRH-R with high potency. We compared the effects of TAL and TRH in competing for [^3^H]MeTRH binding and on stimulating Ca^2+^ release (**Figure 2**). The IC_50_ values were 910 and 36 nM for TAL and TRH, respectively, and the EC_50_ values were 36 and 5.0 nM for TAL and TRH, respectively. Thus, TRH was a high potency agonist and TAL was a moderate potency agonist at TRH-R. We noted, moreover, that the IC_50_/EC_50_ ratio was 25 for TAL (*p* < 0.02), 7.2 for TRH (*p* < 0.05), and approximately 0.4 for MeTRH. When comparing two agonists, the agonist with the higher IC_50_/EC_50_ ratio has a greater intrinsic efficacy (Engel et al., 2006). These findings suggested that TAL may be a more efficacious agonist than TRH and that TRH is more efficacious than MeTRH. We previously showed that TRH exhibited a higher intrinsic efficacy than MeTRH at mouse TRH-Rs (Engel et al., 2006). As TRH is the natural, full agonist, TAL is termed a superagonist, and MeTRH is a partial agonist.

**Table 1:** Pharmacological parameters for TRH-R.

	IC_50_ for binding (nM)	EC_50_ for Ca signaling (nM)
MeTRH	3.0	7.2
TAL	910	36
TRH	36	5.0

**FIGURE 3:**
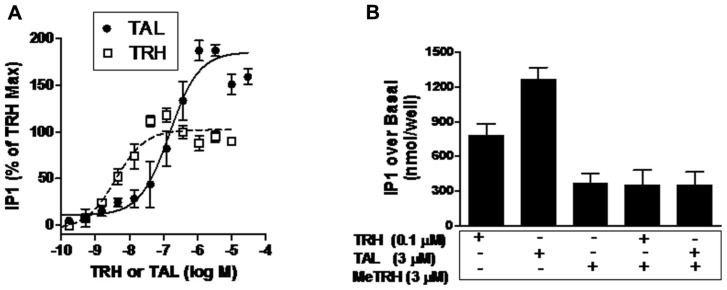
**TAL is a superagonist at TRH-R.** **(A)** IP1 production was determined in HEK 293 cells expressing TRH-R in the presence of increasing doses of TAL or TRH as described in Section “Materials and Methods.” The data represent the mean of duplicate points from five experiments. **(B)** MeTRH inhibits IP1 formation stimulated by TAL and TRH. IP1 production was determined in the presence of TRH (0.1 μM), TAL (3 μM), MeTRH (3 μM), TRH + MeTRH, or TAL + MeTRH. The data are expressed as mean ± SEM performed in duplicate of two experiments.

As Ca^2+^ release is a rapid and transient response to TRH-R activation, it is easier to compare relative intrinsic efficacies by quantifying activation of the inositol-1,4,5-trisphosphate pathway. Inositol-1,4,5-trisphosphate production is the step prior to Ca^2+^ release in signal transduction by TRH-Rs (Gershengorn, 1986) and can be quantified by measuring accumulation of its metabolic product IP1 over time by inhibiting IP1 degradation (**Figure 3A**). The EC_50_ values for IP1 production were found to be 150 nM for TAL and 3.9 nM for TRH. More importantly, TAL was clearly more efficacious than TRH in that TAL stimulated an increase in IP1 production that was 180% of that stimulated by TRH (*p* < 0.001).

Another way of demonstrating relative intrinsic efficacies of agonists is to show that the maximal response of a more efficacious agonist is inhibited by a less efficacious agonist (Engel et al., 2006). **Figure 3B** illustrates the IP1 responses to maximally effective doses of MeTRH, TRH, and TAL. As is evident, TAL (3.5-fold over MeTRH) is more efficacious than TRH (2.1-fold over MeTRH); MeTRH is the least efficacious. As predicted, the least efficacious agonist MeTRH inhibited the response to TRH (full agonist) and to TAL (superagonist).

## DISCUSSION

In this study, we characterized the binding and signaling properties of TAL at TRH-R that, to our knowledge, have not been previously reported. The binding properties of TAL at rodent TRH receptors have been studied previously (Asai et al., 1999; Brown, 1999). In agreement with the findings with rodent receptors, we found that TAL binds to TRH-R with lower affinity than TRH. Our most interesting observation, however, is that TAL exhibits higher intrinsic efficacy than TRH; that is, TAL can stimulate the same level of signaling as TRH but at lower levels of receptor occupancy and could induce higher levels of signaling than TRH at full occupancy (**Figure 3**). Since TRH is the natural, full agonist for TRH-R, TAL is termed a superagonist. We previously reported that other TRH analogs displayed higher intrinsic efficacies than TRH at rodent TRH receptors (Engel et al., 2006). In the same study, we showed that MeTRH, the only TRH analog with higher affinity and potency than TRH, was a partial agonist that displayed lower intrinsic efficacy than TRH and that when high levels of TRH and MeTRH were added simultaneously the level of signaling was lowered to that of MeTRH. This is the predicted effect of adding a partial agonist along with a full agonist. We used a similar experimental design herein to confirm that TAL is a superagonist at TRH-R; MeTRH antagonized IP1 production stimulated by both TRH and TAL (**Figure 3B**).

Previous reports in rodents showed that TAL displayed more activity in stimulating CNS effects than TRH (Suzuki et al., 1990; Brown, 1999). TAL may have similar CNS effects in humans (Gary et al., 2003; Khomane et al., 2011). The differences in the activities of TAL and TRH in the CNS have been attributed to the higher stability in blood and increased penetration of the blood–brain barrier of TAL compared to TRH. Although this is likely true, our new findings of the signaling efficacy of TAL at TRH-R suggest that the higher intrinsic efficacy of TAL may be contributing to its CNS activity in humans also.

In summary, we have described characterization of the pharmacology of TAL at TRH-R. Most importantly, we showed that TAL is a superagonist when signaling at TRH-R via the G_q/11_ protein-phospholipase C-phosphatidylinositol-4,5-bisphosphate-inositol-1,4,5-trisphosphate-calcium pathway. We suggest the superagonism exhibited by TAL may, in addition to its relative metabolic stability and ability to cross the blood–brain barrier, explain its higher activity in mediating CNS effects in humans compared to TRH.

## Conflict of Interest Statement

The authors declare that the research was conducted in the absence of any commercial or financial relationships that could be construed as a potential conflict of interest.
